# Everyone Breathes: a mixed methods evaluation of a combined Feldenkrais and vocal improvisation group within an adult mental health Recovery College setting

**DOI:** 10.3389/fpsyt.2026.1735103

**Published:** 2026-02-19

**Authors:** Megan Cartier, Jhian Cadimas, David Sulkin, Owen Reilly, Joanna Harries, Jenny Hill, Lucas Robson, Sophie Harris, Catherine Elizabeth Carr

**Affiliations:** 1 Research & Development Department, South West London and St George’s Mental Health NHS Trust, London, United Kingdom; 2 National Opera Studio, London, United Kingdom; 3 Recovery College, South West London and St George’s Mental Health NHS Trust, London, United Kingdom; 4 Unit for Social and Community Psychiatry, WHO Collaborating Centre for Mental Health Services Development, Centre for Psychiatry and Mental Health, Wolfson Institute of Population Health, Queen Mary University of London, London, United Kingdom

**Keywords:** breathwork, Feldenkrais method, mental health, mixed methods, Recovery College, vocal improvisation

## Abstract

**Background:**

A range of singing interventions exist for mental health contexts, but few have incorporated group-based body, breathwork and vocal improvisation. The Feldenkrais method has potential to support singing interventions by raising attention to body and breath. This study aimed to describe practice and evaluate experiences and outcomes of a Feldenkrais and group vocal improvisation workshop provided by professional musicians and a Feldenkrais practitioner in a Recovery College setting.

**Methods:**

We used an observational mixed methods design. The workshop was held by a Recovery College for adults living with mental health challenges. All group attendees were invited to participate in the evaluation. Those who consented completed questionnaires at the workshop beginning, end, and two weeks later. We assessed mental wellbeing alongside goals, emotions, group appraisal, un/helpful and important events. Researchers attended as participant observers and conducted a focus group on the third day. We used interpretive content analysis to understand group content and evaluations. Quantitative data were analysed descriptively.

**Results:**

Eight service users (4 male, 5 white British, mean age=43) consented to participate. Group practice was summarised following TIDieR principles and emphasised equality and collaboration. Service users were attracted to the unusual course content, with a strong wish to connect with others and learn. Feldenkrais movement facilitated authentic vocal exploration, expression and creativity. This was experienced initially with some anxiety, but later supported bonding, enjoyment and fun. Experiences were positive, with benefits relating to capacity to breathe, connection to the body, ability to express themselves and find an authentic voice, as well as gain group support. Wellbeing improved on average by 3.14 (range 1–8) to a moderate wellbeing score of 25.9 with a standard deviation (SD) of 2.9. The workshop intensity and course focus were highlighted as areas for future development.

**Discussion:**

The Everyone Breathes workshop offers an intensive, creative, and supportive experience within the Recovery College context. The pilot evaluation suggests the group was well-received, safe and has potential to improve wellbeing in a relatively short timeframe. Future research should explore how to develop content and broaden opportunities for wider implementation to enable formal assessments of effectiveness.

## 1 Introduction

A range of music-based interventions exist to support mental health. These interventions are provided by music practitioners, music therapists and mental health professionals in different public and health care settings. Content can take a variety of forms, from active music-making to listening-based participation and composition. A recent umbrella review estimated medium effect sizes of music-based interventions on mental health outcomes (1), while a meta-analysis found significant effects on both physiological and psychological outcomes of stress (2). Thus, music interventions can aid people in both their personal mental health recovery and wider community participation (3, 4).

Singing-based interventions are one form of music-based intervention (3, 4). Positive effects have been demonstrated for a wide range of populations (5–9). These interventions often take the format of choral participation with the aim of jointly participating in shared songs or performance (9). Groups are commonly led by musicians or music therapists with wellbeing-related goals, as opposed to in-depth emotional exploration. Here, the focus is more upon the activity of group singing improving mood, reducing anxiety/depression and breaking isolation. It is therefore common for these groups to be highly structured, for example, singing groups completing warm-ups and structured rehearsals over a set period of time (10, 11).

Less frequently, groups may incorporate more creative approaches, such as vocal improvisation (12–18). This approach, often led by music therapists, psychotherapists or musicians, tends to feature a warmup, or focus upon breathwork and the body, followed by exercises to build up to shared group improvisation. Client groups tend to be from non-clinical populations, although its potential has been evaluated with adults in community mental health settings (16). Musician-led groups tend to focus upon goals of self-exploration/self-actualisation (13, 18) or team-building (13), while music therapist led groups focus upon more in-depth emotional and psychological processes to address clinical needs (12, 15–17). For example, as a musician-led group, McFerrin’s circle songs (15), focus primarily on the experience of participating. Call and response models are used to build group connections and encourage creativity through improvisation. In contrast, music therapists and psychotherapists use group vocal improvisation with a focus on promoting embodiment and reflection upon emotions arising to enable greater confidence, sense of autonomy and sense of self (12, 14–17). These models of vocal improvisation each recognize the importance of integrating breath and bodily sensations as preparatory exercises to support vocal production. In doing so, benefits are reported to be relaxation, greater freedom of expression, finding one’s own voice, confidence, and emotional transformation (17).

The Feldenkrais method is a form of movement therapy where participants are guided through a series of conscious movements aiming to bring more awareness to the body, breath and self (19, 20). Feldenkrais has been used in physiotherapy settings to help with balance, coordination, and pain management (19, 21). A further study demonstrated equivalent benefits on mood when comparing aerobic exercise to a Feldenkrais group (22).

In mental health settings, one randomised controlled trial examined the influence of Feldenkrais on a sample of individuals with eating disorder diagnoses. The study found that the Feldenkrais group displayed more pleasant body image thoughts compared to a control group (23). More generally, Feldenkrais has been identified as being of potential relevance in psychiatry, but to date, research with wider mental health populations is lacking (24). Research has also found that breathwork has a positive impact on stress and anxiety (25). One study demonstrated that just ten minutes a day of controlled breathing had a positive impact on feelings of stress, anxiety, depression, and wellbeing (26). Feldenkrais has potential to support singing-based interventions through raising attention to the body and breath, however, to our knowledge, it has not yet been explored in this format.

Over the last four years, the National Opera Studio (NOS), and the Recovery College based at South West London & St. George’s Mental Health NHS Trust (SWLSTG), have collaborated to offer vocal improvisation workshops incorporating Feldenkrais at an annual summer school. Informal feedback suggested that these workshops were well-received. The NOS therefore commissioned an evaluation of the workshops. The evaluation aims to formalise the practice that was developed, as well as evaluate their potential from the perspective of Recovery College members and facilitators. This paper reports the overall findings of that evaluation. The main objectives were to describe key elements of the group practice to enable wider research and implementation, evaluate the goals of those who attend the group, evaluate helpful and unhelpful elements to improve practice, identify important moments in the group to help to understand which elements of practice were important to attendees, and evaluate self-reported changes by the group to inform future assessments. Researchers hoped to learn more about the potential impact of the sessions for attendees and the potential to share the practice more widely in the future.

## 2 Methods

### 2.1 Evaluation design

The evaluation was a mixed methods service evaluation using stakeholder group discussions before the workshop, participant-observation of the workshop, questionnaires completed with participants before, immediately and two weeks after the workshop, and a focus group held on the day after the workshop. This range of methods allowed us to capture different voices, both of the facilitators and participants, and for the participants, allowed for flexibility in how they took part. Two of the study team researchers (MC, JC) acted as participant observers, recruiting, assisting with data collection, and running the focus group alongside full participation in the workshop itself. We took this decision to support building trusting relationships, which we felt was especially important given the potentially exposing nature of vocal improvisation and potential vulnerabilities of the group. We took a critical realist perspective, acknowledging the influence of language and culture upon each person’s experiences and understanding of the group (27, 28). Accordingly, our epistemology was contextualist, acknowledging the individual contexts of researchers and participants in shaping knowledge (27). We took an experiential approach to the data, focusing on participants’ and researchers’ individual perspectives and contexts. The service evaluation was reviewed and approved by the Audit team at South West London & St. George’s Mental Health NHS Trust.

### 2.2 Participants and setting

As this was a small, descriptive and evaluative study, we used a convenience sample of Recovery College members who had signed up to participate in the workshop as part of the Recovery College summer school within an NHS mental health trust located in London. Recovery College members were adults living with mental health problems in the community. The course was advertised in a summer school course selection pamphlet. This advertisement can be found in the **Supplementary Material** (see **Supplementary Material 2.1**). The course consisted of two full day workshops, followed by a third day that consisted of a focus group and group picnic run in August 2024. Those who signed up for the workshop were approached by researchers prior to the start of the group to be given an information sheet about the service evaluation. Researchers welcomed questions about the evaluation and ensured that participation in the evaluation was voluntary and did not affect their participation in the group.

### 2.3 Data collection

On the first day of the workshop, group members who wanted to participate in the service evaluation completed a consent form and the initial pre-group assessments. Questionnaires were completed again after the completion of the second day of the course, as well as two weeks after the completion of the workshop.

Pre-group questionnaire: The pre-group questionnaire consisted of general demographic information, including age, gender identity, and ethnicity. Participants were asked to describe their main mental health challenges, circle feelings on feeling chart based on how they were currently feeling, and report if they had previously taken part in this workshop.

Short Warwick Edinburgh Mental Wellbeing Scale (SWEMWBS): The SWEMWBS was used in both the pre-group and two-week follow-up questionnaires. It is a seven-item questionnaire consisting of statements about feelings and thoughts regarding the last two weeks. Answers range from one to five, with one being “none of the time” and five being “all of the time.” It has been validated for use within a mental health population (29–31).

Post-group questionnaire: Participants completed the same feelings chart from the pre-group questionnaire. They were also asked why they chose to participate in the group, if they found the group helpful and/or enjoyable, and how they felt after the group. They were asked to describe the most helpful and unhelpful parts of the course, as well as the most important event from within the course and why. They were also asked to write up to three goals they had going into the course and rate the extent to which they felt they achieved these.

Two-week follow-up questionnaire: Participants completed the SWEMWBS and had the opportunity to provide any final feedback they had for the group.

Researcher observations: Researchers (JC and MC) completed a structured reflection form at the end of each workshop day, detailing the activities and timings of events that had taken place, observations on group process, leadership and reflections on their own feelings and experiences of participating.

Focus group: The focus group was completed on the third day of the workshop and lasted for 90 minutes. This was facilitated by the two participant-observer researchers. The group involved both workshop attendees and facilitators and took place in the same venue as the workshop. The group sat in a circle and consented to the group being audio recorded. A topic guide, modelled on the feedback questionnaires, was developed with input from workshop leaders and the Recovery College lead (see **Supplementary Material 1**). The focus group began with each participant stating a word that they would use to describe the workshop. Discussion continued onto topics including helpful and unhelpful aspects of the workshop, as well as suggested changes or improvements for future workshops.

Considering our subjectivity and the context of the review, our own experience in taking part in the course helped guide us on how to facilitate the focus group and respond with participants. This led to a more empathetic and curious approach in hearing what the participants had to say. We considered our subjectivity as a resource when gathering and analysing the data (32).

### 2.4 Patient and public involvement

To ensure the safety and consideration of needs of those attending the workshop, researchers worked closely with the Recovery College lead and workshop facilitators in both the study and questionnaire design. Some members of the research team had lived experience of mental illness, which we also drew upon when designing our evaluation. In acknowledgement of the Recovery College ethos, we chose not to collect formal mental health diagnoses and instead, developed a range of experiences that were common in mental illness. To aid sharing of feeling states, we used freely available “emoji” pictures. Attention was paid to how to introduce the evaluation so as not to deter members from joining or participating fully in the workshop and included steps to introduce the researchers. We ensured that the researchers were present for the full workshop and follow-up calls to build trust and offering the Recovery College lead as an independent point of contact, should participants encounter issues or concerns.

### 2.5 Data management and analysis

The two participant observer researchers (MC, JC) completed data collection and analysis with the study lead (CC) to support and oversee the analytic process. All had experience of inviting participation and collecting data with mental health populations. This was the first experience for the participant observers of participating in a music-based group, which we felt was important as this was likely to mirror the experiences of wider participants. The study lead held experience as a music therapist and researcher working with mental health populations and experience of participating in group vocal improvisation.

As this was an exploratory evaluation, no sample size calculations or statistical tests were performed. Quantitative data were analysed using SPSS 24. Descriptive statistics (mean/SD or frequencies) were generated for baseline data. For SWEMWBS, items were summed, and raw scores were converted to metric scores (31). Scores were classified into high (>27.5), medium (19.6–27.4) or low (<19.5) wellbeing (32) and meaningful (clinically-relevant) positive/negative change based on a change of 3 points (33).

Audio from the focus group recording was transcribed using an approved NHS transcription company. Qualitative data from the group, as well as researcher observations and questionnaires were anonymised and checked for accuracy before importing into NVivo. In keeping with our aim to primarily describe the group, we used interpretive content analysis to summarise the data. Thie approach enabled us to quickly examine content from the different data sources and facilitated replicability for future evaluations (33). After initial familiarisation with all data sources, we began to code individually. Researchers broke down the content into the pre-defined categories of goals, group practice, important events and why, helpful aspects, unhelpful aspects, reasons for attending, and suggested improvements. Within each of these categories, we initially stayed close to the content of the data, especially for questionnaire data, where wider contextual information was often missing. Workshop content was summarised following the TIDieR format (34) and guidance for reporting music-based interventions (35) from researcher observation forms. This data was supplemented with additional insights from participant questionnaires and focus group discussions. The complete TIDieR table can be found in the **Supplementary Material** (see **Supplementary Material 2.2**).

Initial coding was done independently within the group by three researchers (MC, JC, CC). After initial coding, we met as a group and shared our codes, using Post It notes grouped under the initial categories, referencing the participant and the data source. Together, we refined the codes within these categories, reducing duplicates and grouping similar codes together and renaming them. When there were differences in coding, we discussed it as a group to understand the different perspectives this brought and then agreed how to represent this more fully within the coding frame (33).Where categories implied a process (group practice, important events and why), we took a chronological approach to ordering the codes and began to interpret the main processes being described. We agreed to these main processes as a group and summarised them in text form, along with exemplary quotes. We then began to make inferences, drawing upon the deeper content contained in focus group discussions and researchers’ reflective observations to draw out the links described between specific activities and their impact for participants. Once we had finalised our analysis, we shared this with workshop facilitators for their review and feedback. A simplified summary was also shared with workshop participants with the opportunity to comment further.

## 3 Results

### 3.1 Participants

Of the nine service users attending the group, seven consented to complete the questionnaires and the focus group, one consented only to participating in the focus group, and one chose not to participate in either. Participants were aged on average 43 years (SD = 9.4). Four participants identified as male and three identified as female. Regarding ethnicity, five identified as White British, one as White Other and one as Chinese (**Table 1**).

**Table 1:** Sociodemographic characteristics of participants.

Gender identity	Male	4 (57.1%)
	Female	3 (42.9%)
Ethnicity	White British	5 (71.4%)
	White Other	1 (14.3%)
	Chinese	1 (14.3%)
Age		43 (SD = 9.4)

**Table 2** displays the full list of mental health challenges reported by the participants. Participants reported living with an average of seven mental health challenges each (range 3–11). The most commonly reported challenges (by 6/7 participants) were feeling anxious, depressed, living with past traumatic events, and finding it hard to motivate oneself. Three participants self-reported holding or awaiting a diagnosis of Attention Deficit Hyperactivity Disorder (ADHD).

**Table 2:** Mental health challenges.

Mental health challenge	Number of participants reporting challenge (%)
Feeling anxious	6 (85.7%)
Feeling depressed	
Living with past traumatic events	
Hard to motivate self	
Finding it difficult to manage emotions	4 (57.1%)
Feeling stresses	3 (42.9%)
Feeling guilty	
Intrusive thoughts	
Other – ADHD	
Hearing voices	2 (28.6%)
Hallucinations	
Feeling lonely	
Relationships with other people are hard	
Feeling confused	1 (14.3%)
Feeling scared	
Other – Bipolar	

### 3.2 Workshop content

The course was held over the span of three days at the NOS. Day One and Two ran for five hours with a break for lunch. Day Three consisted of the focus group and a group picnic. The facilitators included a Feldenkrais method facilitator, an opera singer, a cellist, and the studio director of artist development, who has extensive experience in access to arts and education. Two members of the Recovery College staff and two researchers from SWLSTG also participated in the group alongside the nine participants.

Day One: The first day began with introductions within the group. Participants stated their names and were told the loose plans for the workshop. The session began with a Feldenkrais session where participants were guided through to recognise different body parts through slow movements and breathing. This transitioned into the vocal component of the course, which was led by the opera singer. Participants were instructed to stand up and walk around freely while making sounds (such as ooooo, ahhhh). The group was then guided into the next task, which involved small groups writing down words they associate with a particular fruit. These words were then presented to the group. The next activity involved completing an improvisation for a chosen fruit, this time being a lemon. The associated words were “energised”, “summery”, and “healing”. The group made sounds and vocalisations to contribute to the lemon song, where the cellist accompanied. Throughout this session, there was a lot of jokes and laughter. Connections and cohesiveness were both noted as being developed as the group became a safe space for individuals to contribute. After the lunch break, the group reconvened and went through another Feldenkrais session to recentre back into a calm headspace. The group transitioned back into the fruit work and created songs for both nectarines and pineapples. Participants began to introduce new sounds throughout, including non-vocal noises such as tapping or clapping. The last activity of the day involved combining the three fruits and creating a melody with them, where the group transitioned from one to the other. Feedback in final group discussions from Day One included that participants felt immediately welcomed and that the space was inclusive and judgement-free. Members said that it was nice to interact with the group and not have to discuss diagnoses. Group members had different levels of involvement, with some offering feedback frequently and others choosing not to share.

Day Two: Day Two began with a guided Feldenkrais session, then back into the vocal work, making sounds such as “sssss, aaaa, ooooo” and phrases such as “no way, wooowww”. It was discussed how these sounds could be felt in different parts of the body. The group then moved into the fruit work. The first fruit chosen was a grape. To build upon previous methods, group members were each given a grape to look at and feel as they made improvised sounds, accompanied by the cello. This continued with an apple and then a melon. This time, there were no set words to go by as a precursor to sound making, it was “just a matter of starting”. Afterwards, the group discussed this song, with people stating it was harder to come up with sounds without pre-selected words. Someone countered by saying that made it feel easier. It was also discussed how the group came into Day Two with more expectations compared to yesterday as they now knew a bit more about what to expect from the group. After lunch, the group came together and began with the Feldenkrais method. The group transitioned back to the last sound improvisation, where they could taste the fruit prior to the vocalisation. The chosen fruits were a melon and pineapple. Participants did a mixture of walking around, pausing while standing, and sitting down when creating sounds. Some participants walked the whole time, while others would pause, and some sat down. Participants provided feedback stating that since completing the session yesterday, they were at more peace of mind. They reported finding a deeper connection with music, as well as sleeping more the night prior. Participants reported feeling less self-conscious at the end of the course. The session ended with one last sound that consisted of a “fruit salad”. Participants could taste either melon, pineapple, banana, or oranges. Participants laid down with their heads inward and the cellist sat in the middle.

### 3.3 Group objectives

#### 3.3.1 Objective one: Describe key elements of group practice

Key group elements from the “Everyone Breathes” workshop are summarised in the TIDieR table, for example, materials used, approaches and procedures followed (see **Supplementary Material 2.2**). Workshops took on a clear structure but had flexibility in how far and with what focus creative vocal explorations and overall themes were taken. Objects for inspiration were chosen for their sensory qualities (especially touch, taste, smell). Workshops were highly resourced with skilled practitioners for each element (Feldenkreis, vocalisation, instrumental accompaniment, mental health support). One key element identified by the group was that the course was a step-by-step process. The Feldenkrais warm-up allowed participants to connect with the body at its current state, allowing individuals to gradually become aware of the body and its sensations. The transition to the breathwork, and then subsequent songs, allowed individuals to comfortably go from one exercise to the next. Facilitators took time throughout the sessions to check-in with the group and see how the experience was, and how the group wished to continue. When it came to completing the songs, the same gradual approach was used, where new elements were slowly added into practice. This allowed participants to explore each objects’ texture, colour, and other characteristics at a reasonable pace.

There were also no expectations leading into the group. Participants could engage as little or as much as they wanted, allowing everyone to feel comfortable and create their own level of involvement. Participants were given choices throughout the course, such as the choice to sit in a chair, lay down, or sit up on the floor during a song. Participants could offer whatever sounds they wanted to within the group when creating a song, whether it be a vocal sound or a sound made with the hands, and at any volume they were comfortable with. Group decisions led the flow of the course, which allowed participants to create an experience based on what they would like to do.

#### 3.3.2 Objective two: Evaluate goals for attendance

Participants described attending the course for a range of reasons. The most common was an interest in breathwork, either as a novel approach, or due to known benefits of breathwork for mental health. Escaping home difficulties and a need to get out were cited, whilst the length of the course, unusual location and partnership with the NOS made it attractive and stood out to participants in relation to the wider Recovery College courses. Some observed the potential for relaxation or self-discovery and some wished to attend again having attended a previous workshop:

“I think it was the ‘Everyone Breathes’ that really attracted me. But I have to be honest, there wasn’t a lot of info on the poster. I kind of assumed it would be breath work but, you know, National Opera [Studio] that really excited and intrigued me. It was somewhere that wasn’t [hospital] which was a big attraction, a new space and it sounded like something I had never done before and I am trying to challenge myself at the moment even if it makes me feel uncomfortable” [SWL007]

The goals for group attendance fell into two main categories: Social connection and to try something new. All goals were labelled by participants as being either partially or fully met after the workshop.

Social connection: This was the most frequent goal held by participants. Participants listed wanting to “meet others”, “make new friends”, and “work in a group”. Individuals also wanted to “build meaningful connections” within the group, reporting that they wanted “get comfortable in a group setting”.

Try something new: Participants largely wanted to “Put myself out there and try something new” within the course, as well as try new Recovery College courses to “gain new skills and a new perspective”. Goals also related to the course content with participants wanting to learn new ways of breathing based on knowledge that this could help with mental health. Participants reported wanting to challenge themselves and find relief from anxiety, with one participant writing “allow myself to be vulnerable.”

#### 3.3.3 Objective three: Helpful and unhelpful elements

##### 3.3.3.1 Helpful elements

The provision of the workshop being held in a non-mental health venue, the Feldenkrais practice, and the group facilitation skills were cited as key helpful elements for participants to feel safe to experiment and be able to participate in vocal exercises. This led to shared play, enjoyment, humour, and feelings of inclusivity and connection.

The new space was helpful for participants, with it being reported that the NOS was seen as a “blank canvas” and offering “new experiences” as it was not associated with mental health care.

The Feldenkrais method was reported as helpful by many participants. It was described as “meditative”, “healing”, and “relaxing”. It brought a sense of connection to the body, and individuals enjoyed the relaxation and movement it brought about:

“It’s interesting that you monitor your breathing rather than having to change it because with meditation you always have to breathe deeply, and I like that. You can just take yourself as you are and just see what’s happening for you, how you connect and it’s also very relaxing … The usual meditation is a bit new age for me sometimes but this was nice … And it was quite profound you know. I walked in on the first day and my heart rate was stupid levels of high, well and truly into triple digits and by the end of the morning I was down to eighty-four and I don’t normally get that low unless I am lying down or asleep. So it was really profound for me.” [SWL008]

The pacing and support of facilitators was brought up as being helpful, noting that the “guided techniques for various activities” were “well thought out”. One participant stated that the slow build-up of the session was helpful with “challenging oneself to feel vulnerable”. The instrumental accompaniment of the cello was described as both grounding and enabling others to bring their voices into group vocalisations. One participant described the cello as an “anchor” during the songs, saying that they focused in on it when they felt lost during the composition. Participants also reported that the facilitators were very supportive and kind, which helped the participants to feel “more centred and more open” to contribute in the group session. One participant noted that the “tone of voice was impactful while delivering the sessions.”

Participants valued how facilitators set up an inclusive group environment without hierarchies and with a focus upon curious exploration rather than right or wrong:

“There have been times when I’ve struggled to walk outside my front door, so this is in a way was a baptism of fire but a very easy comfortable one in the sense that we were doing it together. Everyone was making themselves vulnerable, it was very inclusive, everyone here was so welcoming and friendly and warm. And I think the fact that there was no hierarchy, everyone was joining in, we are all in this together, that made a huge difference because it wasn’t sort of us and them. I really liked that, yeah.” [SWL007]

The opportunity to explore and sense in the session was reported as liberating from the more didactic elements of healthcare:

“…in mental health we are often told do this and you will recover, don’t do that and you will recover. And actually sometimes just having that opportunity to sense what is actually happening in the moment is more beneficial than sort of forcing something to do or not to do. And I really enjoyed that sort of freedom.” [SWL003]

Themes of self-compassion and accountability were also brought up by participants. The combination of support from the facilitators and pace of the course meant that group members could take their own time based on their own personal needs. The activities also enabled them to participate by reducing internal judgement. Participants reported that the sessions allowed them to overcome their own “inner critic”, and one participant reported that the course allowed them to “connect back to their inner child”. One participant described this process happening through repeatedly challenging themself to contribute with opportunities to then step back:

“And then you come to something like this where it’s like make the weirdest noises you can, be completely kinky, enjoy yourself while you are doing it, and challenge yourself. But you are also learning there are moments where you can take a step back and just listen to everybody else doing that. But you can also trust yourself in making the insertions into the group as well, and that was really exciting as well, being able to trust yourself … And then it was like no you are actually an adult, you can make your own boundaries, you can do your own thing and you can express yourself. And that has been really important.” [SWL004]

Another described the group structure and facilitator attitudes as key to reducing internal judgement:

“My challenge was allowing myself to be vulnerable … with the sort of being fruit, being grapes or melon, it was really, really challenging. I thought you were amazing the way that you built us up to it slowly, everything was okay, there was no right or wrong, you couldn’t make a mistake. And I think when I shared that I had that judgemental voice that I was out of tune, I was discordant, whatever the word, and I was really hard on myself but you made it okay, I felt really held, accepted.” [SWL007]

The novel experience of the class was viewed as helpful, as the unique content of the course put everyone on the same starting ground. The sessions and sound making were described as “unexpected”, “novel”, and “gentle”, but also “impactful”. Participants found the course and vocalisations challenging in a good way, allowing them to explore something new. Enjoyment was noted by a number of participants and was observed in the workshop as moments of playfulness, fun and shared humour. The challenge of participants channeling their own mental health journeys in a private and non-triggering way, using the fruit as a catalyst, become an enjoyable novel experience for all during the shared sound making process:

“Making noises you have never made before it’s, you know you are challenging yourself in ways that then set you up to be able to then communicate those feelings or make you feel a little less daunted about doing so. Because you have had experiences like these and you have been met with such, well just no judgement in doing so, it feels like you are being part of something and it’s been well received by other people. And it’s just an amazing positive and empowering feeling.” [SWL002]

Shared experiences of sound making, including “laughter” were some of the most reported helpful aspects of the sessions. One participant stated “group collaborations created connections” [SWL008]. Participants described a “warm feeling of community” and “feeling of shared composition” offering a “new sensation to connect with others” with “humanity and kindness”.

##### 3.3.3.2 Unhelpful elements

There were fewer unhelpful elements identified, but most related to the course intensity, with some participants finding the length too intense, overwhelming, and tiring. They also reported there not being enough breaks during the course. One participant mentioned wanting a communal lunch to further get to know others, and another participant mentioned wanting more clarity in the instructions during the Feldenkrais sessions. It was also mentioned that participants would like to do introductions again on Day Two as it was hard to remember everyone’s names.

In the focus group, two areas were explored relating to changes or improvements. The first related to workshop intensity, with the suggestion to hold a regular programme over a number of weeks rather than a standalone workshop. The second suggestion related to potential for improvisations to cover more specific emotional and autobiographical links. This prompted lively discussion where members debated the opportunities and challenges in doing so. Participants distinguished between challenges of a focus on emotional expression and immediate benefits of more neutral objects as catalysts for expression:

“I don’t think you would start with emotions. I feel like you need to go up like introduce, like fruit was a great, almost like a good gateway to try something that we are not so emotionally connected to. But then to maybe then progress to then get to a point where we can.” [SWL002]

“I am going to play devil’s advocate here. I am not down with the emotional noises thing. Like I think that it would … like right now I think the fruit was great, maybe some other inanimate objects but probably I would end up screaming and crying.” [SWL004]

#### 3.3.4 Objective four: Important moments

Participants highlighted important events throughout the course relating to specific vocal improvisations, connections made post-improvisation, the group experience of vocalisation, being in the present moment and group support. Participants shared the importance of having a “safe space” to try new activities and “put yourself out there”. In doing so, participants had new experiences and discovered new ways of using their voices, both musically and verbally:

“…it actually made us realise that how sounds actually do matter in our own lives.” [SWL012]

Hearing everyone sound together as one was described as “life-affirming” and listening to this combination enabled participants to stay “in the present moment”. Participants described powerful experiences of the physical effects of sounding together enabling them to find their voice, or experience new ways of connecting to their bodies. Some participants spoke of seeing the activities as a personal challenge to themselves to communicate or interact more:

“There have been times in my life where I have felt like I haven’t had a voice, so you come in and you are suddenly using your voice to actually say something without words and suddenly you are like, oh, I have that ability to do something. And then on the other side there is also learning that sometimes you have a voice even if you are not saying or doing anything and that it’s just as loud and that was really, really profound.” [SWL003]

The combination of careful group leadership, structured activities, and subsequent creative exploration led participants to feel a sense of connection and inclusion that enabled them to open up more in later conversations. These were described as helpful as participants learned from each other and shared aspects of their lives and mental health journey:

“The conversations after we did our fruit compositions was a good way of coming together, being open, honest and vulnerable, and getting to know the staff members and participants. It was the most important for me because I spend a lot of time alone and it gave me that ‘intimate’ connection to other people. It challenged me just to open my mouth and talk and to share my experiences and feelings. I learnt a lot from what other participants said too.” [SWL007]

The final composition was discussed by three participants as being important to finding and validating their voice:

“…it was finding myself and my voice as being a part of something. Being able to be heard, to harmonize, to add value is like being in a conversation in the real world. It’s that my voice matters and even here I have a place.” [SWL004]

#### 3.3.5 Objective five: Self-reported changes

##### 3.3.5.1 Wellbeing

**Figure 1:**
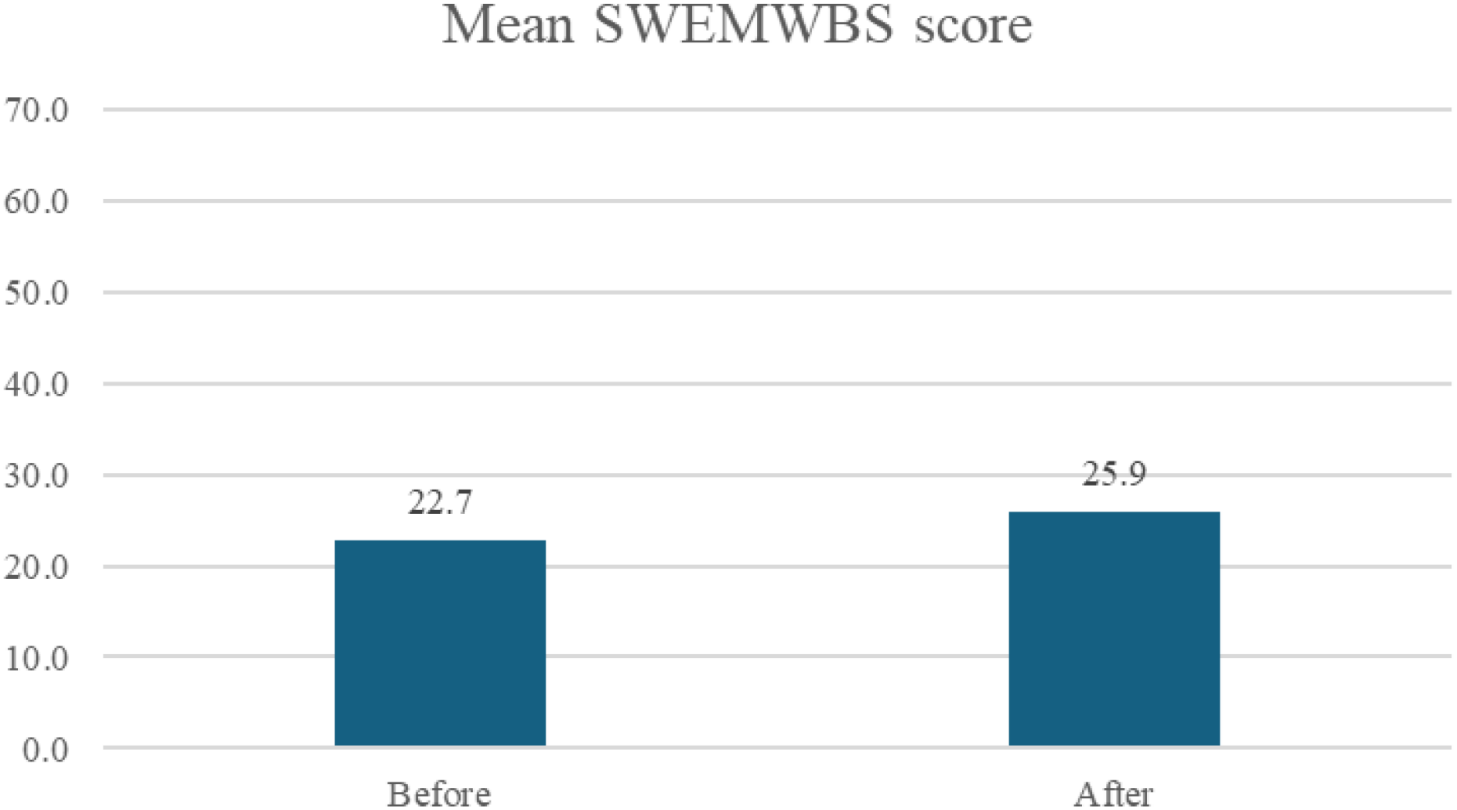
Short Warwick Edinburgh wellbeing scale (SWEMWBS) total scores, before and 2 weeks after the Everyone Breathes workshop. An increase in scores indicates an increase in wellbeing.

Participants’ wellbeing scores improved on average by 3.14 (range 1–8), two weeks after the workshop to an average score of 25.9 (SD = 2.9) (**Figure 1**). This represents a moderate level of wellbeing (scores of 19.6–27.4). Six of the seven participants improved (five from low to moderate wellbeing, one from moderate to high). One participant’s score did not change and remained in the “low” category. Four of the seven participants met criteria for meaningful positive change (change of >3 points). Improvements were seen across all seven items of the scale. Items which had the greatest average change were “feeling useful” (+0.71), “dealing with problems well” (+0.71) and “feeling relaxed” (+0.57).

**Figure 2:**
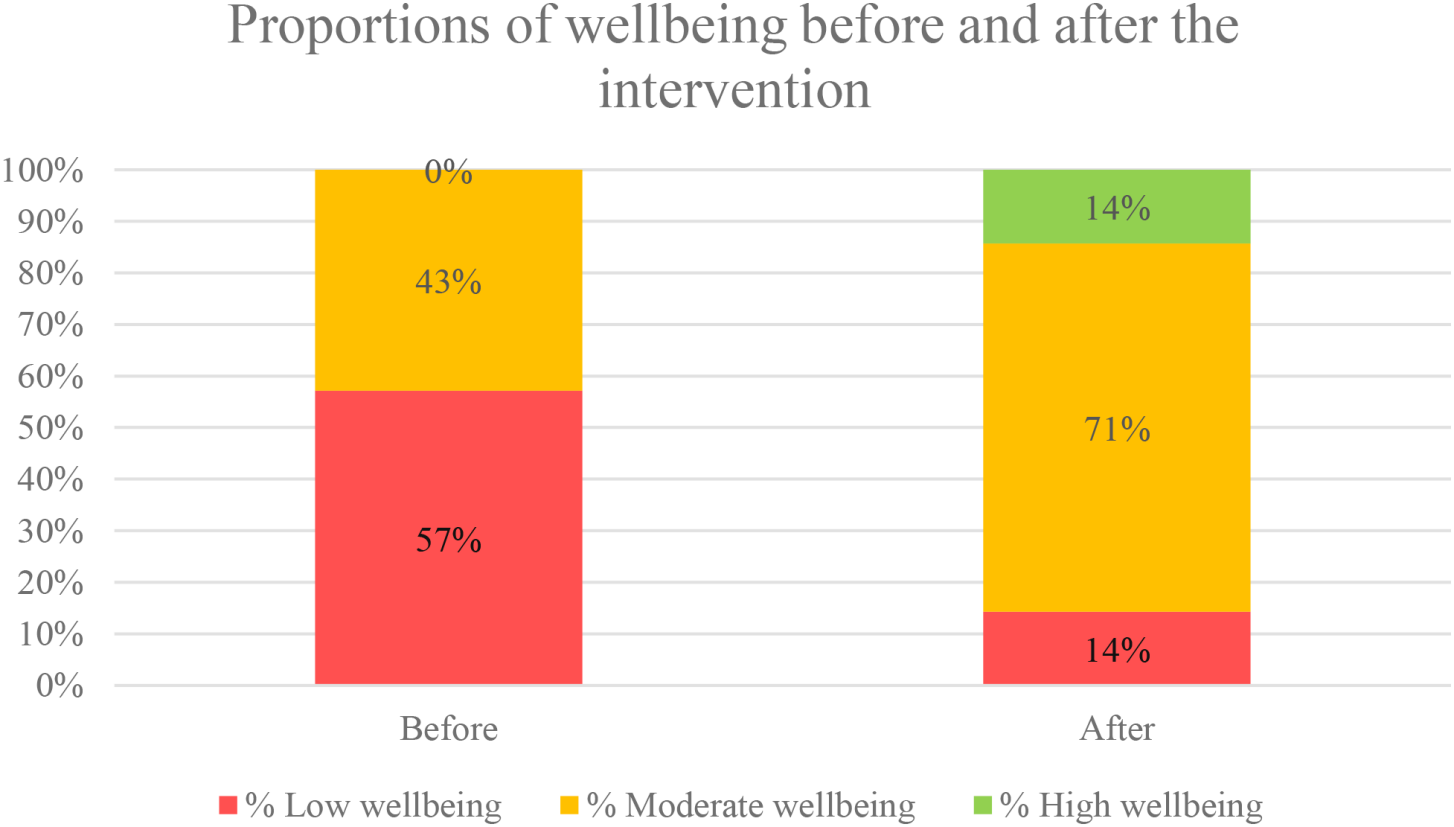
Proportion of participants with low, moderate and high category wellbeing scores before and 2 weeks after the Everyone Breathes workshop. The focus group topic guide, the TIDieR table, and the advertisement used for the course can be found in the **Supplementary Materials**.

Proportions of participants’ wellbeing scores changed showing that low wellbeing decreased, and moderate and high wellbeing increased (**Figure 2**).

##### 3.3.5.2 Course appraisal

All seven participants rated the course as extremely helpful, which was the highest rating available, as well as reporting that they enjoyed the course. Five participants reported feeling much better after the course, and two reported feeling a little better.

##### 3.3.5.3 Emotions before and after the group

**Table 3** displays emotions reported by participants both before and after the group. There was an increase in reports of feeling happy, tired, proud, and relaxed. There was a decrease in reports of feeling excited, stressed, disappointed, sick, lonely and nervous.

**Table 3:** Reported emotions before and after the workshop.

Emotion	N participants pre-group	N participants post-group	Change
Happy	4	6	Increased
Tired	2	5	Increased
Worries	2	0	Decreased
Angry	0	0	
Proud	0	3	Increased
Bored	0	0	
Excited	4	1	Decreased
Upset	0	0	
Embarrassed	0	0	
Sick	1	0	Decreased
Hungry	0	1	Increased
Lonely	1	0	Decreased
Surprised	0	0	
Relaxed	1	4	Increased
Scared	0	0	

## 4 Discussion

This evaluation has provided a detailed account of how the Everyone Breathes workshop was run, alongside experiences of eight service users, the group facilitators and researchers. The group was structured upon a foundation of the Feldenkrais method and breathing, which was carefully built upon with exercises to enable gradual sounding and exploration of the voice. Facilitation drew upon values of equality, coproduction, and empowerment. Service-users attended due to the unusual and attractive nature of the course (being creative, using breathwork, the location being a non-mental health site, and involving the NOS), and with a strong wish to connect with others and learn.

Participants experienced the workshop positively, with many benefits shared relating to capacity to breathe, connect to the body, express vocally, find an authentic voice and gain social and group support. These benefits were realised due to the careful curation and facilitation of the group, led by cofacilitators who emphasised equality, were open to all contributions, and were experts in their relevant fields (Feldenkrais method, opera singing, instrumental accompaniment, creative directorship, mental health support). The curation of this safe nonjudgmental space meant participants felt safe to experiment both vocally and with what they shared. The subsequent creative and collaborative experience of group vocalisation led to very different and profound experiences in participants’ sense of themselves. Whilst primarily designed with creative and artistic goals in mind, five out of the seven participants reported clinically relevant increases from low to moderate wellbeing two weeks later and four reported changes >3 points, suggesting meaningful positive change (31). All participants rated the workshop positively and no adverse or harmful events were reported. The changes suggested were overall simple to consider and implement (communal lunches, more breaks). The workshop intensity and course focus were highlighted as areas for further discussion for development.

### 4.1 Strengths and limitations

This evaluation was completed with information from eight out of the nine workshop attendees, meaning that most, but not all experiences from the workshop are represented. Participants were fairly evenly split between male and female but were generally older and predominantly of white ethnicity. In keeping with the ethos of the Recovery College, we did not collect formal mental health diagnoses, meaning that it is not possible to identify the extent to which dominant psychiatric illnesses were represented. However, all participants reported challenges that could be considered common within secondary mental health services. Three participants independently volunteered confirmed or pending ADHD diagnosis. Future evaluations should explore how to broaden the demographic reach of attendees, particularly in relation to ethnicity and age as well as broader learning and neurodevelopmental conditions, education and wider sociodemographic characteristics. This is important given mental illness, lower socio-economic status and being of non-white ethnicity are characteristics identified as barriers to participating in the arts (36). Loneliness and social contact may be particularly important to capture given the majority of participants sought social connection as a goal in attending this workshop. It is also important to note the short follow-up period of the study. As the follow-up was done two weeks after completion of the course, we are not able to comment on any long-term impact of course involvement.

The small number of participants meant that we were not able to make any generalisations about the overall effectiveness of the group. Similarly, given the potential for group composition to vary, future groups may be received differently, depending on those attending. Further evaluations using the same measures will aid comparability. While no adverse events or harms were apparent during this evaluation, we did not actively ask about these beyond inviting comments on “unhelpful” factors. Feedback may have been influenced by the relationships formed by researchers with participants during the workshop. However, having researchers as participant observers was in keeping with the ethos of both the workshop and research methodology and meant that a more nuanced understanding could be drawn from the feedback. Researchers were able to experience the workshop first hand and build relationships with participants which may have also assisted in building trust and retaining participants for the two-week follow-up.

### 4.2 Comparison with the literature

The findings from this evaluation map closely onto existing models that explain the benefits of participatory music making on wellbeing, namely through managing and expressing emotions, facilitating self-development (including experiences of accomplishment, promoting agency and confidence), and providing a form of respite and facilitating connections (37). The changes observed in this evaluation appear comparable or greater than other studies that have evaluated wellbeing in similar populations (38–41). To note, these studies have longer intervention and follow-up periods, suggesting that the gains observed here warrant further investigation. The workshop also offered a number of factors facilitative of engagement for people with mental illness: Activities focused on building upon perceived capability, providing opportunities for social interaction and reinforcing motivations to engage (42). The techniques that we mapped in this workshop have previously been used within wider group vocalisation courses that have been developed in various forms since the 1970s (12–18). Features shared across all of these approaches include the use of a warm-up, or a main focus upon breathwork and the body, to then build up to shared group vocalisation. It is not clear to what extent Feldenkrais methods are distinct from or overlap with wider breathing and bodywork techniques, although its application in psychiatry is beginning to be explored (24). Our evaluation found that participants valued these techniques as a means of grounding and preparing their bodies for vocal work, thus further exploration of the role and function would be of benefit to understanding the group offer in the future. Similarly, while use of the cello was experienced as grounding and helpful by participants, relatively little information was gathered on how the cello was used moment to moment in improvisations. Further musical analysis of the workshops would be of benefit to understanding group processes and the instruments’ grounding role.

The integration of breath and body-based work was suggested as a key factor in generating safety to explore freely. Breathwork has been found to have a small to medium effect upon stress in nonclinical populations although the exact nature of its effects, particularly in mental health requires further research (25, 26) with proposed mechanisms including modulation of the autonomic nervous system to activate the parasympathetic nervous system – the system that enables the body to relax. There is now a wide body of evidence to suggest beneficial effects of singing upon a range of physiological, psychological, behavioural and social factors (3). Riabzev and colleagues (17) outline key benefits of vocal improvisation as requiring no vocal training, reducing anxiety through breathing exercises, providing a playful space to experience the present moment, promote creativity, enhance recognition and expression of feelings and needs and enable an “intimate, safe and reliable” musical environment. These findings are all mirrored in the results of this evaluation. Similarly to this workshop, healthy participants in one (17) study reported the workshops creating an “open space for freeing and exploring one’s voice”, changing participants’ attitudes towards their own voices and enabling meaningful experiences and new self-discoveries” (17).

The Everyone Breathes workshop was noted for its accessibility by its participants. This is important due to the many barriers faced by people living with mental illness in accessing and participating in arts and wider cultural activities (43, 44). Guidance on the provision of arts in health initiatives within the NHS emphasizes the importance of high quality, non-clinical space (45).The provision of the groups by an esteemed arts organization, the NOS, in a non-NHS mental health setting was appealing to group attendees and mirrors feedback from similar arts-based studies located in conservatoire contexts (11, 46, 47).

Finally, the feedback regarding workshop length and focus offers some pertinent questions for any future Everyone Breathes groups. Participants shared the value of what they had gained in just two days of attendance but also noted challenges in the intensity of this. At the same time, participants were curious as to how these techniques might be used to explore more emotionally evocative and personal issues over time relating to their mental health and recovery. Some expressed concern in doing so, noting the potential for the explorations to become uncontained or “triggering” whilst others felt that the current format was powerful in and of itself. When reviewing findings with the workshop facilitators, the idea of a series of regular workshops to build confidence to join (for example) wider choirs was proposed, along with the potential to empower participants to run workshops themselves. Pujol Torras (16) found an increased need for structure and active therapeutic stance when comparing group musical improvisation with vocal improvisation in secondary mental health community patients and participants also noted the importance of a neutral and abstract topic in feeling safe and able to engage in vocal explorations. The care taken by the group to explore possibilities in this focus group discussion underscores the complexity and potential vulnerabilities requiring careful consideration.

### 4.3 Future directions

This evaluation highlights the importance of the inclusion of the arts in mental health care. The feedback on the Everyone Breathes course was positive and suggests that this type of workshop should continue to be offered within a Recovery College context. Such an offer is in keeping with priorities from WHO (48), UK Government, Arts Council England and the National Centre for Creative Health in prioritizing creative health for all (49, 50). In order to do this, we emphasise the importance of continued clear, unambiguous advertisement of the workshops and high-quality facilitators with the appropriate qualifications to facilitate the group. We recommend ongoing evaluation using the tools from this report to aid comparability.

There is potential to develop workshops in order to meet different stages of the mental health journey and to encourage participation from a wider demographic. Workshops have the potential to be developed into a longer program of work to develop content and relationships or incorporate end products, such as involvement of a composer and professional recording to facilitate a performance and/or sound recording or other artistic artefacts. This approach would enable development of a performative element and have the potential for attendees to have a product with which to demonstrate achievement and remember the experience.

While the workshops were provided by specialised practitioners, there is also potential to adapt the intervention to be led by people with less specialised skills. For example, none of the vocal exercises, warm-ups or improvisations were based on opera classical music and could be led by other trained singers or vocalists. There is also potential in this course to grow and open to larger group sizes, as well as be integrated into community services. Once such developmental work is complete, through example, further case or feasibility studies, formal testing of effectiveness will be possible in a randomised controlled trial.

We recommend coproducing and evaluating these developments with service users in the Recovery College alongside music therapists, dance movement psychotherapists and wider arts therapists, clinical psychologists and psychiatrists to ensure psychological safety.

## 5 Conclusion

The Everyone Breathes workshop is a unique offer within a Recovery College context. The groups are well received, safe and have potential to improve wellbeing within a secondary mental health population. The combination of Feldenkrais bodywork, vocal improvisation and inclusive leadership appears to support experiences of authentic vocal expression and group belonging. There is scope for further development of the workshop to widen participation from people with non-white backgrounds, include an end product or become a longer programme of work. Similarly, there is potential for the workshop to be developed for broader populations, which would align with Arts Council England’s strategic aims to increase access to the health benefits of the arts for all (45). Coproduction with service users, music therapists and wider mental health professionals is recommended for any future developments.

## Data Availability

The raw data supporting the conclusions of this article will be made available by the authors, without undue reservation.
