# Supplementary material for: Everyone Breathes: a mixed methods evaluation of a combined Feldenkrais and vocal improvisation group within an adult mental health Recovery College setting
Source: Front Psychiatry. 2026 Feb 19;17:1735103. doi: 10.3389/fpsyt.2026.1735103 (PMC12960531; doi:10.3389/fpsyt.2026.1735103)
Supplement: Supplementary file 1 [file DataSheet1.pdf]

Supplementary Material

1 Supplementary Data

1.1 Focus group topic guide

This evaluation aims to investigate participants' experiences of the Everyone breathes summer workshop. This topic guide has been designed to obtain the views of participants and group leaders who attended the summer workshop on 12-14 August 2024.

Aims:

- To explore experiences of attending the Everyone breathes group
- To identify factors found helpful within the group
- To identify factors found unhelpful within the group
- To explore suggested changes or improvements

Researchers to tick potential probe questions if they are covered during the discussion and then follow-up with probes which are not covered.

1.1.1 Introduction

- Researchers introduce themselves- acknowledge that you have got to know each other during these workshops.
- Reminder that this is part of an evaluation to help to understand what works well in these workshops and what can be improved
- Provide assurances about confidentiality
- Stress that personal disclosures are not expected
- Explain what happens to data collected- transcribing, reported, anonymity
- Check how group would like to be referred to during the recording
- Introduce audio recorder
- Explain how focus group works – moderator will not say very much and will tend to ask questions. All views are important. No right or wrong answers. Looking for a range of views, consensus not required.
- To say if they are feeling stressed/uncomfortable. Do not have to answer a question or can change topic if needed.
- Mobile phones off or on to silent
- Invite any questions

1.1.2 Background

- Ask each person in the group to say their name and one word they would use to describe their experience of the Everyone breathes workshop
- Potential probes
  - Why did you choose that word?
  - How did the workshops do that?
  - Can you link that word to a specific activity or event from the workshop?

1.1.3 Helpful features of the workshops

- What was helpful about attending the Everyone breathes workshop?
  - Referral into the workshop
  - Administration before the workshop began
  - Location
  - Group leaders
  - Other participants
  - Feldenkreis warm up
  - Vocal warm up
  - Professional musician supporting

1.1.4 Unhelpful features of the workshops

- What was unhelpful about attending the Everyone breathes workshop?
  - Referral into the workshop
  - Administration before the workshop began
  - Location
  - Group leaders
  - Other participants
  - Feldenkreis warm up
  - Vocal warm up
  - Professional musician supporting

1.1.5 Suggested changes or improvements

- How can we change the workshop to improve it in the future?
  - Referral into the workshop
  - Administration before the workshop began
  - Location
  - Group leaders
  - Other participants
  - Feldenkreis warm up
  - Vocal warm up
  - Professional musician supporting
  - Length
  - Other ideas?

1.1.6 End of workshop

- Any final thoughts or ideas- anything they would like to add

- Thank participants for their time
- Remind re: confidentiality and recording
- Confirm re: sending summaries for review / future contact permission

2 Supplementary Figures and Tables

2.1 Advert placed in Recovery College Summer School brochure for Everyone Breathes

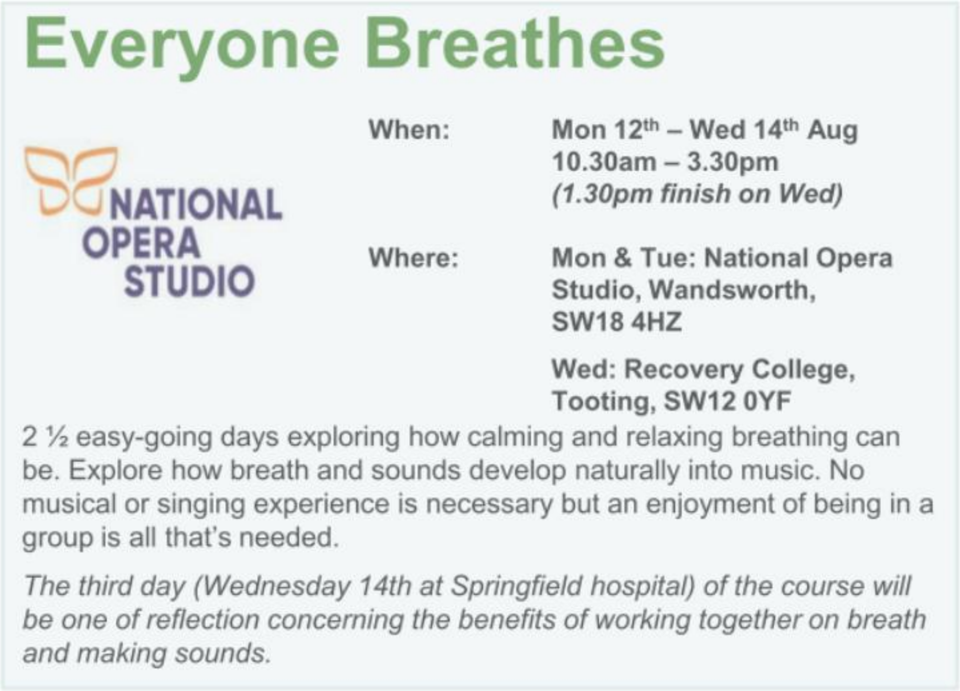

2.2 Summary of the Everyone Breathes workshop following the TIDieR template for reporting complex interventions.

TiDier item	
1. Name of group	Year 1 Workshop: All sounds allowed/aloud<br>Year 2 Workshop: Fabric of sound (performed in year 3)<br>Year 4 Workshop: Everyone Breathes
2. Why this approach?	Workshops were commissioned to provide a creative option to do with singing for the Recovery College. The Everyone Breathes workshop was designed to transition from breath work to 'sounding,' expression, and eventually vocal and social confidence with the aim of enabling participants to increase confidence in their exploration of breath, vocalisation and expression in a non-judgmental way, access wider community singing, such as choirs

	as a social, therapeutic and musical activity and to connect, in each participant, the importance, value and celebration of using breath and subsequently sound as part of the notion of 'recovery'.<br><br>Breath 'is the stuff of life'. It is key to our ability to relax and calm. It is also key to vocalising our main means of communication. Making sounds with the voice is the pathway to singing.<br><br>Activities were designed to focus on visualising the breath and then use this in guided exercises to explore feelings and ideas. Feldenkrais method was incorporated due to its dual focus on body and breath, and guided gentle movements to promote body awareness and anchor participants.<br><br>**Feldenkreis warm-up** lying down with floor for support, check-ins allow participants to connect to their bodies, reduce tension and critical mind to reach a freer, more simple state. Freedom to participate creatively and without judgement. Returning to Feldenkreis throughout the workshops works like an anchor to help people stay connected to their bodies.<br><br>**Breath to vocalisation:** Gradual movement to build confidence in using breath and voice in new and different ways; builds a toolkit/creative palette of options for later improvisational work.<br><br>**Instrumental accompaniment**: Addition of cello provides a focus on a sound which then frees up participants not to feel like they are at the centre of the improvisation.<br><br>**Group improvisation** demands working together simultaneously and active and careful listening of group members to each other. Focus upon group process rather than individual skills supports members who lack confidence in their voice to participate.<br><br>Nonjudgement of sounds reduces self-criticism, meaning less likely to be judgmental of others. Equality of direction, ideas and contributions, across all participants (facilitators/participants) emphasized.
3. What materials were used?	Flowers, snacks and refreshments (tea, coffee, biscuits) available to access at any time when needed during the group. Cello as a grounding instrument. Chairs, yoga mats and pillows were available to use. Objects as creative stimulus: Selection of different items with a theme for example, types of fabric (100+)/Objects from nature/Different types of fruit

4.	What procedures were used? How were the days structured?	**Connecting with the body** (Feldenkrais practitioner): Warm-up with Feldenkreis methods. Tune into the body where it is at, noticing and being without judgement how you feel, body weight, breath. Introduce simple movement to connect to body and then to breath.<br><br>**Breath to sound:** Opera singer takes through gentle exercises to move from breathing into sound, starting with box breathing/numbered breathing moving next to unsounded consonants (sss,hhh,shhh) to percussive consonants (ttt,bbb,ddd) and then sounded vowels and consonants (mmmm,aaah). Invitations to vary volume in either a single mode (eg. Quiet) or dynamic (going from quiet to loud). Check in after each exercise- was that easier or harder? Keeping body focus- where did you feel this in your body? Exploring use of body.<br><br>**Move into freeing up use of sound:** Group facilitators may model by making extreme/exaggerated strange sounds. Group are invited to offer a new or different soundbonus points for making it as strange or weird as possible.<br><br>**Movement into sustained vocalisation:** Instrument (cello) is brought in for a sustained ground note. Time taken to listen to the note of the cello – where do you feel in the body? Invitation to join or find another note or sound. Explore going up and down. This often leads to group harmonisation (crunchy, soupy).<br><br>**Time allowed for group reflection, breaks where needed.**<br><br>**Move into more creative work with objects as a stimulus. Introduce idea of visualisation.**<br><br>**Fabric/fruits/objects-** if they were sounds what would they be like?<br><br>**Group are free to determine where to take this exploration.**<br><br>**End reflection.**
5.	Did you have any guiding principles as to how you led the workshop?	- Always checking in after every section– anything to share?<br><br>- Gentle instructions<br><br>- Invitations to explore<br><br>– All sounds are allowed, no right or wrong sounds

	- Exploring experience at the end of improvisations – how was that? How did you feel?<br><br>- Feeding back elements of discussions into the next improvisation.<br><br>- Promoting equality and democracy in the group. Facilitators participate in all activities and are led by group suggestions.<br><br>- Giving options of ways people can choose to participate in the improvisation (different roles, options, leading, following, subgroups, listening) harmony/melody.<br><br>- Use of Feldenkrais to return to body after a break or beginning a new section of work.<br><br>**Instrumental accompaniment:**<br><br>-Use of cello – range and frequency are close to vocal range, bodily resonance. "Offering" a bass/ground. Initial single note grounding to 'anchor' to go into sounds/singing, gradually building from simple bass lines to more complex. ---Musically staying 'out of the way' but responding to what is heard.<br><br>- Avoid formal musical terms.<br><br>**Group facilitation**<br><br>-Implicit ground rules- everyone respectful of contributions and welcoming of others in the group. If you need time out or toilet you are welcome to take a break/leave at any point.<br><br>-Facilitators model positive feedback on the sounds created. What does this sound like? (no correct answer).<br><br>-Freedom to move in the group space as wished (eg. Moving into subgroups in the physical space gave some confidence to vocalise)<br><br>-Responding to suggestions made by the group shares power with participants and allows them to take ownership of creative process.
6. Who provided it? What were their roles?	**Feldenkreis practitioner:** Feldenkreis exercises and breathwork<br><br>**Mezzo-soprano:** Vocal exercises and improvisations<br><br>**Cellist:** Instrumental grounding and later accompaniment/co-improvisation in vocal improvisations.

	**Composer (workshop 2 held in 2021-2022 only):** Create graphic score, assist with recording of performance.<br><br>**Recovery college lead: provide** mental health support to participants if needed during workshop.<br><br>**Director of Studio:** Designed overall workshop concept. Ensure balance and progress in the<br><br>workshops; direct contributions of creative practitioners. Act as a link and facilitator between the group participants and facilitators. Monitor timings and direction of the workshop.<br><br>All cofacilitators are active participants in all elements of the activity (as opposed to observers). Seamless transition between planned elements.
7. How was it provided? (e.g. group or individual)	Group
8. Where did it take place?	National Opera Studio, Wandsworth
9. When and How much? (Number, frequency, duration of sessions)	Standalone workshops<br><br>Workshop 1: 1 day<br><br>Workshop 2: 3 days, break, further 3 days and a performance the following year<br><br>Workshop 3: 2 days plus half day feedback
10. Tailoring	Plans could be adapted on the day based on group direction of participants. Eg. Suggestions to do exercises in a circle. Feldenkreis- option to do exercises seated rather than lying down.
11. Modifications	Workshop 1: The piano was used as accompanying instrument. After workshop 1, cello preferred over piano for its single line qualities, closeness to vocal range and capacity to still ground.<br><br>Workshop 2: Longer duration (3 days, break, 3 days): Fabric of sound Group settled on 6 pieces of fabric of different tactile qualities with each representing different types of sounds. A graphic score was created to aid the group to transition between sections but remain together while also learning how to listen carefully to one another to make this transition. The composition

	was recorded and shared between participants. A performance was held at an annual exhibition by Hospital Rooms the following year.<br><br>Used the word improvisation rather than singing<br><br>One workshop used dimmed lights on suggestion by the group
